# Agenesis of Isthmus of the Thyroid Gland in a Patient with Graves-Basedow Disease and a Solitary Nodule

**DOI:** 10.1155/2013/608481

**Published:** 2013-01-02

**Authors:** Omer Faruk Ozkan, Mehmet Asık, Huseyin Toman, Faruk Ozkul, Oztekin Cıkman, Muammer Karaayvaz

**Affiliations:** ^1^Department of General Surgery, Faculty of Medicine, Canakkale 18 March University, Canakkale, Turkey; ^2^Department of Endocrinology and Methabolic Disease, Faculty of Medicine, Canakkale 18 March University, Canakkale, Turkey; ^3^Department of Anestesiology, Faculty of Medicine, Canakkale 18 March University, Canakkale, Turkey

## Abstract

The thyroid is a vascular endocrine gland with two lateral lobes connected by a narrow, median isthmus. Although a wide range of congenital anomalies of the thyroid gland has been reported in the literature, agenesis of the thyroid isthmus is a very rare congenital anomaly. Thyroid isthmus agenesis does not manifest clinical symptoms, and it can be confused with other thyroid pathologies. We describe a patient with no isthmus of the thyroid, associated with Graves-Basedow disease. Thyroid isthmus agenesis should be kept in mind in order for surgical procedures involving thyroid pathologies to be carried out safely.

## 1. Introduction

The thyroid gland consists of two lobes connected by an isthmus, which also includes thyroid tissue. There are numerous studies investigating the length and depth of the thyroid isthmus by a means of age and evaluating the relationship between reference values and thyroid disorders [1]. The incidence of isthmus agenesis, referring the total absence of the isthmus of the thyroid gland, is not fully known, and the condition is reported through case reports and cadaver studies. 

The most common cause of hyperthyroidism is Graves-Basedow disease. Hyperthyroidism usually accompanies with diffuse goiter, ophthalmopathy, and skin changes [2]. There are reports of thyroid developmental anomalies such as single lobe hemiagenesis accompanying Graves' disease [3]. We present a case of absence of the thyroid isthmus associating with Graves-Basedow disease.

## 2. Case Report

**Figure 1:**
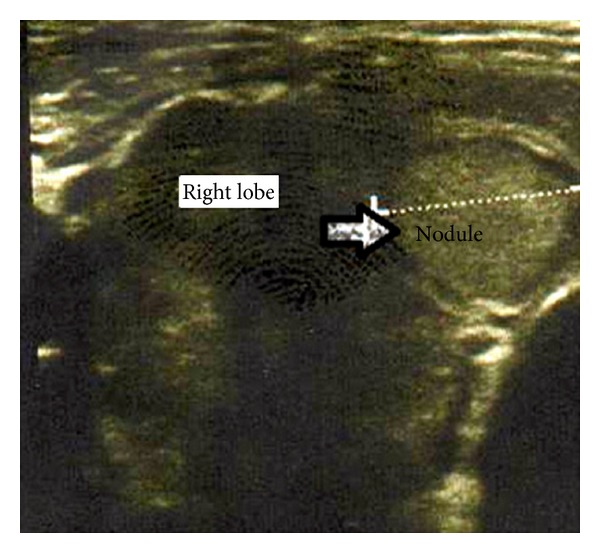
Ultrasonography of thyroid gland showing heterogeneous parenchyma and the solitary nodule in right lobe.

**Figure 2:**
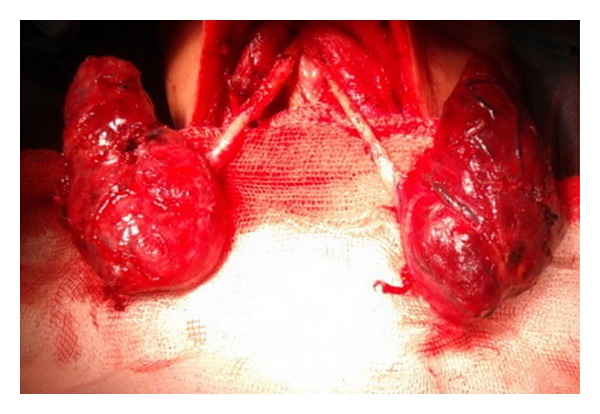
Agenesis of the thyroid isthmus at surgical exploration.

A 41-year-old woman was admitted to the endocrinology department with a history of palpitation, weight loss, myalgia, shortness of breath, and dyspnea during exertion for the previous 2 weeks. The history revealed a previous Graves' disease and vitiligo for the last 14 years. She had used propylthiouracil and propranolol at irregular intervals. Her background was unremarkable, apart from a history of hypertension and smoking for 24 years. The patient had no thyroid surgery. Physical examination revealed tachycardia, minimal exophthalmos, diffuse palpable goiter, and prevalent vitiligo on the face and body. Laboratory tests were Hb: 8.3 mg/dL (11,7–15,5 mg/dL), hct: 24.8% (34,5–46,3), TSH: 0.005 IU/mL (0,270–4,2 IU/mL), FT4: 3.2 pmol/L (0,93–1,7 pmol/L), FT3: 12.2 pmol/L (2,0–4,4), Anti-TPO: 600 IU/mL (0–34 IU/mL), AntiTG: 4000 IU/mL (0–115I U/mL), and vitamin B12: 82.4 pg/mL (191–663 pg/mL). Ultrasonography of the thyroid showed heterogeneous parenchyma, an 11-mm nodule in the right thyroid lobe (Figure 1). Thyroid scintigraphy revealed increased iodine uptake. On admission, she had not been using her medications for the previous 15 days because of agranulocytosis. In medical management, Lugol's solution 3 × 5 drops/day, propranolol 3 × 40 mg/day, and dexamethasone 3 × 2 mg/day were given. When she was euthyroid, a total thyroidectomy was scheduled. In surgery, operative findings confirmed that the right and left lobes of thyroid gland were completely separated due to isthmus agenesis (Figure 2). A total thyroidectomy was performed as a standard procedure. Histopathological examination of specimen revealed a patchy lymphocytic infiltrate and mild thyroid hyperplasia, which is characteristic of the Graves' disease and benign colloid nodule in right lobe.

## 3. Discussion

 The isthmus of the thyroid gland contains normal thyroid tissues, with a dimension of 1.25 cm transversely and vertically [1, 4]. Isthmus agenesis of the thyroid gland is an uncommon developmental anomaly. According to a cadaveric study, isthmus agenesis is seen 5 fold more in males. [4]. Although the incidence varies from 3% to 10% in different studies, the true figure is uncertain [5–7], possibly because of no-symptom population. This is diagnosed only when there is a symptom, such as nodular goiter, thyroiditis, or primary carcinoma [8]. 

The underlying etiology of isthmus agenesis has not been well defined. Genetic factors and defects in embryological development seem to play an important role in thyroid isthmus agenesis, mutations in the genes responsible for the development of the thyroid may be associated with isthmus agenesis, especially TITF1-2 genes [9] and chromosome 22 [10]. In embryogenesis, the thyroid gland begins to develop in the early days of gestation. The thyroglossal duct grows from endodermal thickening in the floor of the pharynx at the level of second and third pharyngeal pouches. It descends to its final location anterior to the trachea and bifurcates to form the two thyroid lobes connected by a median isthmus [5–8]. As described by Pastor Vázquez et al. [8], a fusion anomaly of the thyroglossal duct in the midline leads to two independent lobes with no isthmus of the thyroid gland.

 Thyroid gland may have various developmental anomalies. To the best of our knowledge, this is the first case report of isthmus agenesis in the English literature, associated with Graves-Basedow disease with a solitary nodule. Due to its rare nature, isthmus agenesis should be kept in mind for safe surgery to avoid complications during neck operations, and rare but possible coincidence with Graves' disease that also has a thyroid nodule.
